# Supplementary material for: A whole-animal phenotypic drug screen identifies suppressors of atherogenic lipoproteins
Source: eLife. 2026 Aug 27;14:RP105314. doi: 10.7554/eLife.105314 (PMC13521566; doi:10.7554/eLife.105314)
Supplement: Supplementary file 2 [file elife-105314-code1.zip › Output_data/Enoxolone_SL.pdf]

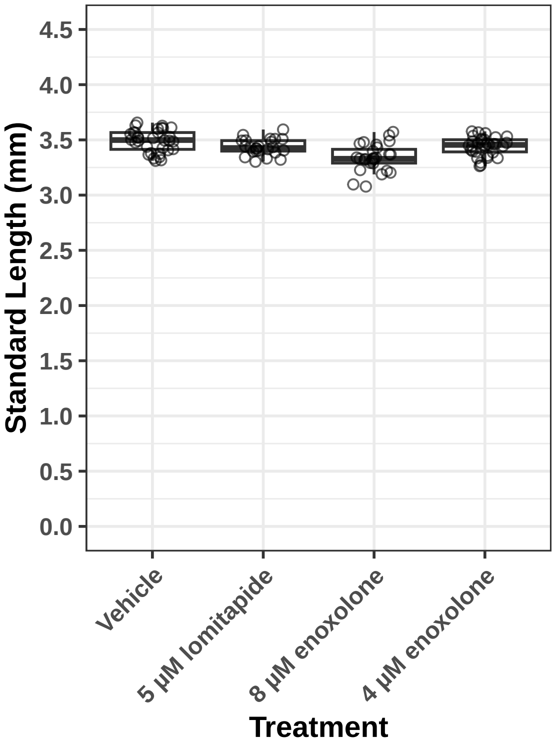

Standard Length (mm)
4.5
4.0
3.5
3.0
2.5
2.0
1.5
1.0
0.5
0.0
Vehicle
5 μM lomitapide
8 μM enoxolone
4 μM enoxolone
Treatment
